# Transforming Growth Factor-β1 T869C Gene Polymorphism Is Associated with Acquired Sick Sinus Syndrome via Linking a Higher Serum Protein Level

**DOI:** 10.1371/journal.pone.0158676

**Published:** 2016-07-05

**Authors:** Jan-Yow Chen, Jiung-Hsiun Liu, Hong-Dar Isaac Wu, Kuo-Hung Lin, Kuan-Cheng Chang, Ying-Ming Liou

**Affiliations:** 1 Division of Cardiology, Department of Medicine, China Medical University Hospital, Taichung, Taiwan; 2 Department of Life Sciences, National Chung Hsing University, Taichung, Taiwan; 3 School of Medicine, China Medical University, Taichung, Taiwan; 4 Department of Applied Mathematics and Institute of Statistics, National Chung Hsing University, Taichung, Taiwan; University of Alabama at Birmingham, UNITED STATES

## Abstract

**Background:**

Familial sick sinus syndrome is associated with gene mutations and dysfunction of ion channels. In contrast, degenerative fibrosis of the sinus node tissue plays an important role in the pathogenesis of acquired sick sinus syndrome. There is a close relationship between transforming growth factor-β1 mediated cardiac fibrosis and acquired arrhythmia. It is of interest to examine whether transforming growth factor-β1 is involved in the pathogenesis of acquired sick sinus syndrome.

**Methods:**

Overall, 110 patients with acquired SSS and 137 age/gender-matched controls were screened for transforming growth factor-β1 and cardiac sodium channel gene polymorphisms using gene sequencing or restriction fragment length polymorphism methods. An enzyme-linked immunosorbent assay was used to determine the serum level of transforming growth factor-β1.

**Results:**

Two transforming growth factor-β1 gene polymorphisms (C-509T and T+869C) and one cardiac sodium channel gene polymorphism (H588R) have been identified. The C-dominant CC/CT genotype frequency of T869C was significantly higher in acquired sick sinus syndrome patients than in controls (OR 2.09, 95% CI 1.16–3.75, P = 0.01). Consistently, the level of serum transforming growth factor-β1 was also significantly greater in acquired sick sinus syndrome group than in controls (5.3±3.4 ng/ml vs. 3.7±2.4 ng/ml, P = 0.01). In addition, the CC/CT genotypes showed a higher transforming growth factor-β1 serum level than the TT genotype (4.25 ± 2.50 ng/ml vs. 2.71± 1.76 ng/ml, P = 0.028) in controls.

**Conclusion:**

Transforming growth factor-β1 T869C polymorphism, correlated with high serum transforming growth factor-β1 levels, is associated with susceptibility to acquired sick sinus syndrome.

## Introduction

Sick sinus syndrome (SSS) is a group of abnormal heart rhythm disorders resulting from malfunctions of the sinus node. The prevalence of SSS is about 1/600 in the population with age over 65 years. Over half of the global pacemaker implantations were performed for the SSS patients [1, 2]. The electrophysiological abnormalities of SSS patients include sinus node arrest, sinus node exit block, profound sinus bradycardia and bradycardia-tachycardia syndrome [1, 2]. Evidence also indicated that genetic mutations in ion channels might lead to familial SSS [3–6]; however, limited information is available regarding the pathophysiologic mechanism of age-related acquired SSS [7, 8]. Acquired SSS, most commonly observed in the elderly, results from degenerative fibrosis and aging sinus node tissue [2, 6]. Apparently, variations in the genes encoding proteins involved in fibrosis and aging would affect the susceptibility to acquired SSS [8]. The spontaneous depolarization of the sinus node has been attributed to the inward current driven through sodium (I_Na_), calcium (L-type Ca^2+^ current, I_Ca,L_; and T-type Ca^2+^ current, I_Ca,T_) and funny currents (I_f_). In addition, the voltage-gated sodium channel, type V alpha subunit (SCN5A) gene encoding the cardiac sodium channel has been shown to trigger the action potential of the sinus node [6]. I_Na_ plays an important role in pacemaking and action potential conduction, particularly in the periphery of the sinus node [6]. In addition, SCN5A mutation or variation has also been associated with cardiac arrhythmia, sinus node dysfunction, and conduction disorders [9, 10]. However, it remains unclear whether alterations in SCN5A gene expression contribute to acquired SSS.

Transforming growth factor β1(TGF-β1), a member of the transforming growth factor family, has been associated with cell growth, proliferation and differentiation, tissue fibrosis, tumor progression and metastasis, wound healing, embryogenesis and immune modulation [10–13]. In addition, TGF-β1 involved in the renin-angiotensin system (RAS) plays a role in cardiac fibrosis [12]. A study using the SCN5A knockout mouse model showed that TGF-β1-mediated fibrosis and ion channel remodeling could lead to sinus node dysfunction [10]. Clinical studies also reported that the TGF-β1 serum level is highly associated with the risk of atrial arrhythmia [14, 15]. However, a role for TGF-β1 in the pathogenesis of age-related acquired SSS has not been determined. Nevertheless, studies have shown that TGF-β1 gene polymorphisms modulating TGF-β1 serum levels [13, 16, 17]. Thus, we hypothesized that TGF-β1 gene polymorphisms might exert a significant effect on sinus node function and the susceptibility to acquired SSS via linking TGF-β1 serum levels.

## Materials and Methods

### Ethics statement

The protocol of present study was approved by the Institutional Review Board of China Medical University Hospital (DMR-IRB2-158) in Taiwan. Written informed consent was obtained from each participant or each participant’s guardian after a full explanation for the present study. The investigation conformed with the principles outlined in the Declaration of Helsinki.

### Subjects

During the period from August 1, 2012 to July 31, 2013, a total of 110 consecutively eligible patients with documented SSS and a control group of 137 subjects matched for age and gender in frequency were included for the present study. All of the SSS patients and controls are Taiwanese population. Medication such as digitalis and antiarrhythmic drugs that can modify the heart rate had been discontinued for at least five half-lives before observing the heart rate. Acquired SSS was diagnosed based on evidence of irreversible symptomatic bradycardia with a sinus long pause of greater than 3 seconds or profound sinus bradycardia of less than 40 beats/min for more than 1 min while awake [18, 19]. The sinus pauses and bradycardia were recorded by a series of resting 12-lead electrocardiogram (ECG) and ambulatory ECG recorders. All SSS patients met the indications for permanent pacemaker implantation.

All control subjects were randomly selected from cardiovascular outpatient department and ward and were frequency-matched to SSS patients by gender and age at the same hospital during the same period. Patients with history of reversible bradycardia, familial SSS, serious systemic disease, unstable angina, acute myocardial infarction or acute myocarditis were excluded.

### Genotyping and association study

Blood samples from 137 controls and 110 SSS patients were prepared to isolate genomic DNA using a commercially available DNA extraction kit (Illustra^TM^, GE Healthcare). A final reaction of 50μL mixture including 100 ng genomic DNA, 2–6 pmol of selected primers, 1X Taq polymerase buffer, and 0.25 units of AmpliTaq Gold^TM^ polymerase (Roche) was prepared for Polymerase chain reactions (PCRs). A programmable thermal cycler (GeneAmp PCR system 2700, Applied Biosystems, CA) was used for PCRs. The PCR cycling conditions were set with 1 cycle at 94°C for 5 minutes, followed by 35 cycles at 94°C for 30 seconds, 56°C for 30 seconds, and 72°C for 60 seconds, with a final extension cycle at 72°C for 7 minutes. The primers for the TGF-β1 C-509T (rs1800469) polymorphism were 5'-CGCAACTTCGACCGCTAC -3' (forward) and 5'-CTCGGCGACTCCTTCCTC-3' (reverse). The primers for TGF-β1 T+869C polymorphism (rs1800470) were 5’-TTCCCTCGAGGCCCTCCTA-3 (forward) and 5’ -GCCGCAGCTTGGACAGGATC-3’ (reverse). The primers were designed according to the sequences of the human TGF-β1 gene (GenBank accession number: J04431) and TGF-β1 precursor gene (exon 1; X05839) [13]. The primers for the SCN5A polymorphism A1673G (H558R, rs1805124) were 5’-GCGAGACCTGGGTTCTGA-3’ (forward) and 5’-GTGGCTTCCTGGGGATGTT-3’ (reverse). The quality of the PCR products was assessed through the electrophoresis of 4 μL of the PCR reactions on a 3% agarose gel containing ethidium bromide. Genotyping of the TGF-β1 gene C-509T and T+869C polymorphisms was determined through DNA sequencing. A gene sequencing analyzer (ABI 3730 XL DNA Analyzer, Applied Biosystems) was used to determine the gene sequences of the PCR products. Genotyping of the H558R polymorphism was performed through restriction fragment length polymorphism (RFLP) analysis as described in detail previously [20]. The corresponding A to G nucleotide transition creates the sequence -CCGC-, which is the recognition site for the restriction enzyme *AciI*. This variant, located in exon 12 of SCN5A, was first amplified within a 251-base pair (bp) PCR product. Restriction digestion of the PCR product using *AciI* resulted in the following fragment sizes: H allele(-CCAC-): 201 and 50 bp; and R allele (-CCGC-): 181, 50 and 20 bp. The fragments were separated using agarose gel electrophoresis. Hence, the H and R alleles could be distinguished based on the presence of 201 and 181bp fragments, respectively.

An association study between the gene polymorphisms and acquired SSS was performed via measuring the frequency of the alleles, genotypes and haplotypes of the TGF β1and SCN5A polymorphisms in the acquired SSS and control groups.

### Measurement of the serum TGF-β1 concentration

Serum samples were randomly obtained from 44 of the 110 acquired SSS patients and 49 of the 137 control individuals. The samples were stored at -20℃ until further analysis. Serum TGF-β1 levels were detected using the enzyme-linked immunosorbent assay kit (ELISA) (Invitrogen, CA, USA). The detection minimum of this assay was 15.6 pg/mL, and the intra-assay and inter-assay coefficients of variation (CVs) were 5.5 and 7.5%, respectively. The sera were activated through acidification/neutralizing and tested at a 1:40 dilution. The optical density (OD) was measured using an ELISA Microplate Reader set to 450 nm (VersaMax, Molecular Devices, Sunnyvale, CA, US). The samples were run in duplicate technical repeats for the serum protein levels. The TGF-β1 concentration was determined using a standard curve according to the manufacturer’s instructions.

### Quantification of TGF β1 mRNA expression

A centrifuge (Kubota) was used to isolate the peripheral blood mononuclear cells (PBMC) for TGF-β1 mRNA isolation. mRNA samples were randomly obtained from 15 of the 110 acquired SSS patients and 18 of the 137 control individuals. Real-time quantitative polymerase chain reaction (qPCR) was used for quantitative measurement of mRNA expression of TGF-β1. TRIzol® Reagent (Invitrogen) was used to isolate total RNA based on the manufacturer’s protocol. Reverse transcriptase (M-MLV-RT, Genemark), oligo-dT and 2 μg of total RNA was used for reverse transcription. SYBR qPCR Kit (Genemark) and an ABI Prism 7300 sequence detection system (Applied Biosystems) were used for quantitative analysis of mRNA level. qPCR was run in duplicate for each sample. SYBR Green dye was used as a real-time reporter of the presence of double-stranded DNA. The primers specific for TGF-β1 were listed as followings: forward, 5’- CCCAGCATCTGCAAAGCTC-3’, reverse, 5’- GTCAATGTACAGCTGCCGCA-3.

### Statistical Analysis

Continuous data were expressed as the means ± standard deviation (SD). Student’s t test was used for the continuous data with normal distribution. Mann-Whitney U test was used for the continuous data without normal distribution. Conventional chi-squire test was used for comparison of categorical data if the observation numbers in all categories were larger than 5; otherwise, the Fisher exact test was replaced for data analysis. One-way analysis of variance (ANOVA) was used for comparison among numeric variables for the TGF-β1 and SCN5A genotypes. The χ2 goodness-of-fit test with 1 degree of freedom was utilized to exam the deviation of the genotype distribution from Hardy-Weinberg equilibrium (HWE) for each TGF-β1 or SCN5A polymorphism. Haploview software was used to analyze the haplotype frequency of the gene polymorphisms [21]. The gene polymorphisms were likely not separated through recombination process and linkage disequilibrium (LD) might be developed. The LD between the polymorphisms of TGF-β1 was analyzed via pairwise measurement of LD. In addition, owing to the D’ magnitude varies according to the sample size; the *r*^2^-value was used to confirm the LD. We also utilized expectation-maximization (EM)-based haplotype frequency estimation with a permutation test to confirm whether a specific haplotype was associated with acquired SSS [22, 23]. D’>0.7 and.*r*^2^>1/3 were defined as statistic significance of the LD [23, 24]. *P* values less than 0.05 were considered statistically significant.

## Results

### Patient characteristics

The clinical characteristics of the SSS patients and controls were summarized in Table 1. Baseline characteristics were similar between groups.

**Table 1:** Clinical characteristics of study patients.

	SSS	Control	
	(N = 110)	(N = 137)	p
Age (years)	70.3±11.1	70.2±9.1	0.898*
Gender (male/female)	34/76	46/91	0.656^§^
Heart rate (beats/min)	45.9±11.5	75.2±13.6	<0.0001
PR interval (ms)	175.3±33.0	161.6±25.3	0.002
HT (n, %)	47 (42.7%)	62 (45.3%)	0.691 ^§^
DM (n, %)	25 (22.7%)	27 (19.7%)	0.563^§^
CAD (n, %)	11 (10.0%)	13 (9.5%)	0.893 ^§^
AF (n, %)	22 (20.0%)	24 (17.5%)	0.62 ^§^

*Mann-Whitney U test

^§^χ^2^ test; SSS = sick sinus syndrome; HT = hypertension; DM = diabetes mellitus; CAD = coronary artery disease; AF = atrial fibrillation

### HWE test and LD analysis

**Fig 1:**
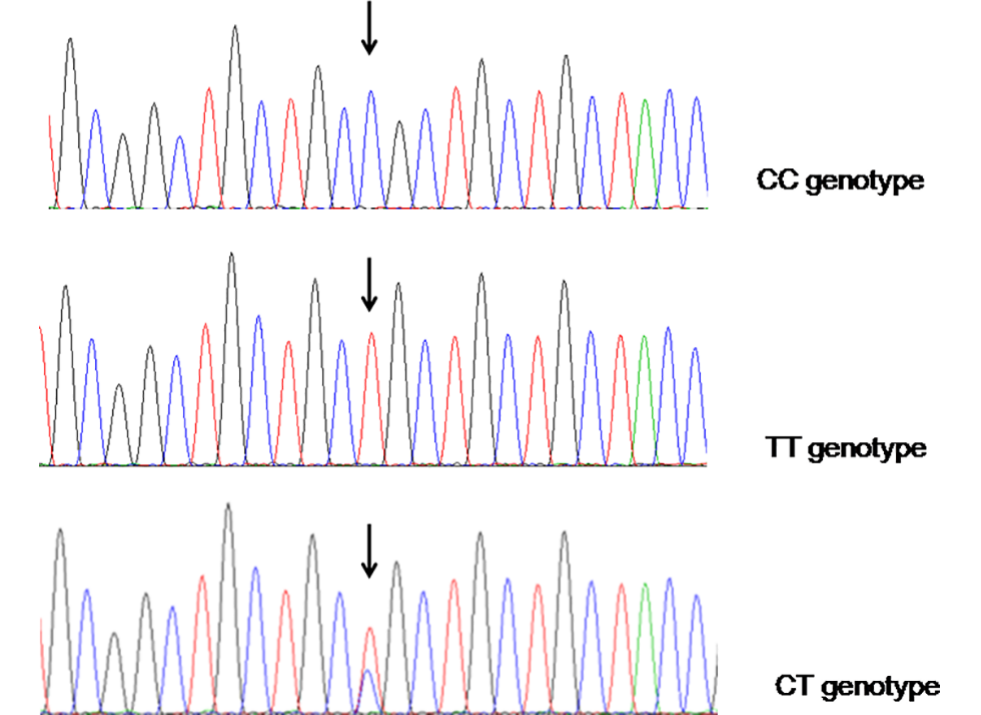
Gene sequencing for the different genotypes of the TGF-β1 T869C polymorphism. The polymorphic sites of CC, TT and CT genotypes were indicated by the arrows.

In the present study, two polymorphisms at positions -509and +869 in the TGF-β1 gene (Fig 1) and the polymorphism H558R (A+1673C) in the SCN5A gene were observed. HWE test was utilized to exam the deviations of the genotype counts of each polymorphism. The P-values were 0.09, 0.22 and 0.18 for C-509T, T869C and A1673G gene polymorphisms, respectively. We also used the HWE test to exam the deviations of TGF-β1and SCN5A genotype distribution in the SSS and control groups respectively. In control group, the P-values were 0.36, 0.75 and 0.76 for C-509T, T869C and A1673G gene polymorphisms, respectively. In SSS patients, the P-values were 0.15, 0.085 and 0.087 for C-509T, T869C and A1673G gene polymorphisms, respectively.

We used the LD test with D’ and r^2^ to assess the pair-wise linkage between the two polymorphic sites of TGF-β1 gene. The D’ value of the -509/+869 loci pairs was 0.87 and the corresponding r^2^ value was 0.556. The D’ and r^2^ values indicated a significant but not complete linkage at the loci pair -509/+869, reflecting the distribution of haplotypes for the TGF-β1 gene.

### Gene association studies

The genotype distribution at position +869 was significant different between SSS and controls (Table 2, P = 0.04). The C-dominant CC/CT genotype frequency of T869C was significantly higher in acquired SSS patients than in control individuals (OR 2.09, 95% CI 1.16–3.75, P = 0.01). There was also a significant difference in the distribution of allele frequency at position +869 between subjects in the SSS and control groups (P = 0.026). The C allele frequency of TGF-β1 was significantly higher in the SSS group than in the control group (OR = 1.5, 95% CI: 1.049–2.45, P = 0.026) (Table 2). No significant difference in the genotype and allele frequency distribution was observed in TGF-β1 C-509T and SCN5A H558R polymorphisms between the subjects in the SSS and control groups (P>0.05).

**Table 2:** Genotype and allele distribution of TGF β1 and SCN5A in SSS patients and controls,

Gene polymorphism	Genotypes and Alleles	SSS patients	Control patients	p
		(N = 110)	(N = 137)	
**SCN5A gene**				
A1673G	AA–n (%)	101 (91.8)	130 (94.9)	
	AG–n (%)	8 (7.3)	7 (5.1)	
	GG–n (%)	1 (0.9)	0 (0)	0.423
	A:G–n (%)	210 (95.5):10 (4.5)	260 (94.9):14 (5.1)	0.772
**TGF beta 1 gene**				
C-509T	CC–n (%)	19 (17.3)	28 (20.4)	
	CT–n (%)	62 (56.4)	74 (54.0)	
	TT–n (%)	29 (26.4)	35 (25.5)	0.841
	C:T–n (%)	100 (45.4):120(54.5)	130 (47.4):144(52.6)	0.659
T+869C	CC–n (%)	24 (21.8)	22 (16.1)	
	CT–n (%)	64 (58.2)	68 (49.6)	
	TT–n (%)	22 (20.0)	47 (34.3)	0.04
	CC+CT/:TT–n (%)	88 (80.0):22 (20.0)	90 (65.7):47 (34.3)	0.01
	C:T–n (%)	112 (50.9):108 (49.1)	112 (40.9):162 (59.1)	0.026

SSS = sick sinus syndrome. The upper *P* value is for comparison of genotype frequencies and the lower is for allele frequencies.

### Haplotype analysis

Four haplotypes in the TGF-β1 gene were identified to examine the relationship of these variations with SSS (Table 3). The TT haplotype (-509T,+869T) occurred at a significantly lower frequency in the SSS group than in the control group (haplotype frequency: 0.0627 vs. 0.1380; OR 0.418, P = 0.0066; Table 3). The TC haplotype (-509T, +869C) showed a significantly higher frequency in the SSS group than in the control group (haplotype frequency: 0.4827 vs. 0.3803; OR 1.521, P = 0.0221).

**Table 3:** Haplotype frequency of the TGF β1 gene polymorphisms in SSS patients and controls.

Haplotype						
		Overall	SSS	Controls		
-509	+869	(N = 247)	(N = 110)	(N = 137)	OR	P*
C	T	0.442	0.4282	0.4533	0.903	0.5761
T	C	0.426	0.4827	0.3803	1.521	0.0221
T	T	0.105	0.0627	0.1380	0.418	0.0066
C	C	0.028	0.0263	0.0285	0.924	0.8889

* There results were confirmed through permutation test. TT is the only significant candidate haplotype (P = 0.0226).

### Serum TGF-β1 protein levels in the various TGF-β1 polymorphisms

**Fig 2:**
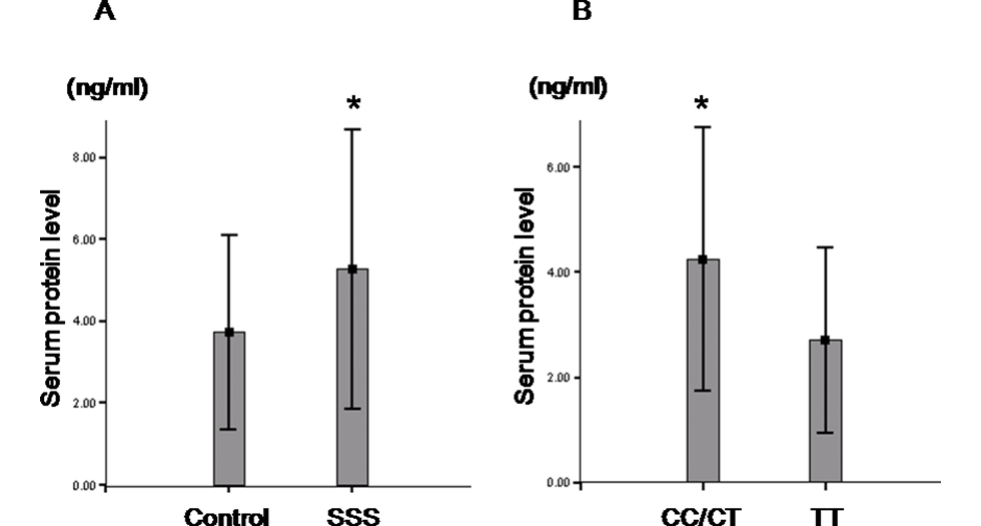
Serum TGF-β1 protein levels. (A) The samples were randomly collected from 49 controls and 44 SSS patients. The serum TGF-β1 protein level in SSS patients (N = 44) is significantly higher than that in control individuals (N = 49). (B) The samples were all from control group (N = 49) including 32 cases with CC/CT genotypes and 17 cases with TT genotypes. The serum TGF-β1 protein level in controls with a CC/CT genotype is significantly higher than in those with a TT genotype. *<0.05.

ELISA revealed that the level of TGF-β1 in the serum was significantly greater in the SSS group (N = 44) than in the control group (N = 49) (5.3 ± 3.4 ng/ml vs. 3.7 ± 2.4 ng/ml, P = 0.01) among randomly selected subjects. The 44 SSS patients included 35 patients with CC/CT genotypes and 9 patients with TT genotypes. The 49 controls included 32 subjects with CC/CT genotypes and 17 subjects with TT genotype. In addition, the CC/CT genotype (N = 32) showed a higher TGF-β1 serum level than the TT genotype (N = 17) (CC/CT vs. TT = 4.25 ± 2.50 ng/ml vs. 2.71 ± 1.76 ng/ml, P = 0.028) in the control group (N = 49) (Fig 2).

### TGF-β1 mRNA expression levels of the various TGF-β1 gene polymorphisms in SSS patients and controls

**Fig 3:**
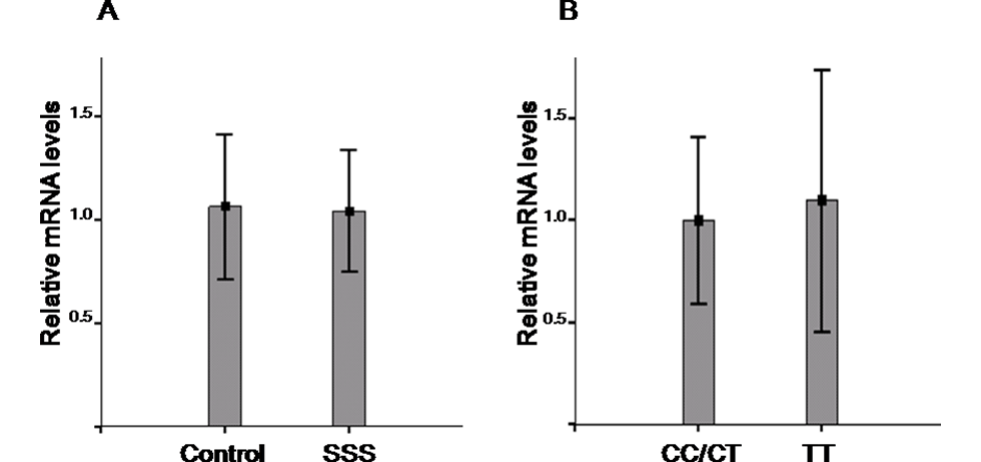
TGF-β1 mRNA expression levels in different genotypes of SSS patients and controls. (A) Quantitative analysis of TGF-β1 mRNA expression using GAPDH mRNA as inner control revealed there is no significant difference in the TGF-β1 mRNA expression levels between SSS patients (N = 15) and control individuals (N = 18) (relative mRNA expression levels, SSS vs. control = 1.05 ± 0.27 vs. 1.06 ± 0.35 P = 0.976). (B) The samples were all from control group (N = 18) including 11 cases with CC/CT genotypes and 7 cases with TT genotypes. There is no significant difference in the TGF-β1 mRNA expression levels between CC/CT and TT genotypes (relative mRNA expression levels, CC/CT vs. TT = 0.98 ± 0.40 vs. 1.07 ± 0.63, P = 0.899). In (A) and (B), each value represents the mean ± SEM.

The samples were randomly collected from 18 controls and 15 SSS patients. Quantitative analysis of TGF-β1 mRNA expression revealed there is no significant difference in the TGF-β1 mRNA expression levels between SSS patients (N = 15) and control individuals (N = 18) (relative mRNA expression levels, SSS vs. control = 1.05 ± 0.27 vs. 1.06 ± 0.35 P = 0.976) (Fig 3A). The 15 SSS patients included 10 patients with CC/CT genotypes and 5 patients with TT genotypes. The 18 controls included 11 subjects with CC/CT genotypes and 7 subjects with TT genotype. There is also no significant difference in the TGF-β1 mRNA expression levels between CC/CT and TT genotypes in the controls (relative mRNA expression levels, CC/CT vs. TT = 0.98 ± 0.40 vs. 1.07 ± 0.63, P = 0.899) (Fig 3B). The results indicates that the TGF-β1 T869C gene polymorphism could not significantly affect the TGF-β1 mRNA expression.

## Discussion

The present study demonstrated the C-dominant CC/CT genotype frequency of TGF-β1T+869C polymorphism is significantly higher in the SSS group. The TGF-β1 serum protein level was significantly higher in the CC/CT genotypes than in the TT genotypes in the control group. The serum TGF-β1 protein levels were also higher in SSS patients than in the controls. These data suggested that CC/CT genotypes associated with higher TGF-β1 protein levels in serum might be associated with disease susceptibility to acquired SSS.

### Aging and acquired sick sinus syndrome

Studies in animal models have shown that TGF-β1-mediated fibrosis and ion channel remodeling play key roles in the sinus node dysfunction linked to SCN5A-deficiency and aging.[10] The loss of connexin-43 expression and the down-regulation of L-type calcium channels (I_ca,L_) have been associated with sinus node dysfunction in aging [25, 26]. The down regulation of sodium and potassium channels has also been linked to sinus node aging process [27, 28]. These findings suggested that atrial remodeling with fibrosis and the down regulation of cardiac ion channels and gap junction proteins are important factors in the pathophysiological mechanism of aging-dependent acquired SSS.

### TGF-β1 affecting sinus node function via linking the RAS molecules

Acquired SSS has been attributed to cardiac fibrosis during aging process and the modification of ion channels through RAS [8, 29]. The overexpression of RAS downregulates the expression of sodium channel and gap junction proteins, leading to low-voltage electrical activity and conduction in the heart [30]. Studies using transgenic mice demonstrated that the overexpression of angiotensin II type I (AT1) receptor in myocardium is associated with sinus bradycardia [31]. These results indicate that overexpression of RAS molecules might result in fibrosis, ion channel modification and sinus node dysfunction. TGF-β1 has been associated with the RAS and cardiac fibrosis [12]. In vitro studies have shown that angiotensin II might upregulate TGF-β1 expression through the mitogen-activated protein kinase (MAPK) signaling pathway via the AT1 receptor in cardiac fibroblasts, myofibroblasts, and myocytes [12]. A TGF-β1 autocrine/paracrine cellular response induced through the binding of angiotensin II to the AT1 receptor has been observed in cardiac fibroblasts [12]. These studies indicate a critical role of TGF-β1 in the activation of the RAS in fibrosis and the modulation of ion channels, resulting in the dysfunction of the pacemaking activity in fibrotic heart tissue.

### Gene polymorphisms and susceptibility to acquired SSS

Information concerning the influence of gene polymorphisms on the pathophysiological mechanisms of acquired SSS is limited [7, 8]. A recent genome-wide association study reported that a rare variant in the MYH6 gene, which encodes the alpha heavy chain subunit of cardiac myosin, is associated with a high risk of acquired SSS based on the population of Iceland [7]. This study showed that a rare missense variant, C2161T, results in the conceptual amino acid substitution Arg721Trp, associated with acquired SSS. The variant C2161T is considered to be linked to cardiac-specific microRNA and gap junction protein expression, and subsequently affects sinus node function [7]. This study proposed differential mechanisms for acquired and familial SSS. However, acquired SSS is also a complex disease with a collection of manifestations of sinus node dysfunction rather than a single etiology and pathogenesis [32, 33]. Notably, the complex susceptibility of acquired SSS might be determined through multiple susceptibility loci, ethnic population, environment factors and age differences.

In a previous study, we demonstrated that the angiotensinogen (AGT) promoter polymorphism G-6A is highly associated with susceptibility to acquired SSS.[8] Based on functional studies, including electrophoretic mobility shift assay (EMSA) and luciferase assay results, we confirmed that nucleotide G at position -6 of the AGT gene modulates binding affinity with nuclear factors and yields lower transcriptional activity than nucleotide A. The G-6A AGT promoter polymorphism modulates AGT expression and therefore associated with acquired SSS. The results indicate that the pathophysiologic mechanism of acquired SSS is associated with the RAS [8].

In the present study, we fist reported that TGF-β1 T869C gene polymorphism is associated with the disease susceptibility to acquired SSS. It is likely that TGF-β1 T869C gene polymorphism could affect the sinus node function via linking to the modulation of TGF-β1 protein levels in serum.

### Gene association study of TGF-β1 T869C gene polymorphism with SSS associated cardiovascular diseases

AF, CAD and HT have been reported to be associated with acquired SSS [2, 32, 34]. The results of the present association study between the TGF-β1 T869C gene polymorphism and disease susceptibility of acquired SSS might be influenced by these cardiovascular diseases. We have performed analysis by doing the same association study for AF, CAD and HT before/after excluded known SSS patients. Before excluded SSS patients, no significant difference in genotype distribution (CC+CT/TT) of TGF-β1 T869C gene polymorphism was observed between the subjects with and without AF (34/12 vs. 144/57, OR = 1.122, 95% CI 0.543–2.318, p = 0.757), CAD (19/5 vs. 159/64, OR = 1.530, 95% CI 0.548–4.271, p = 0.417) or HT (74/35 vs. 104/34, OR = 0.691, 95% CI 0.396–1.208, p = 0.195). After excluded SSS patients, there was also no significant difference in the genotype distribution (CC+CT/TT) between the subjects with and without AF (14/10 vs. 76/37, OR = 0.682, 95% CI 0.277–1.679, p = 0.405), CAD (9/4 vs. 81/43, OR = 1.194, 95% CI 0.348–4.105, p = 1.000) or HT(39/23 vs. 81/43, OR = 0.789, 95% CI 0.393–1.6915, p = 0.532). The results of the association study indicate that TGF β1 T869C gene polymorphism is not associated with AF, CAD or HT. The results also support that TGF β1 T869C gene polymorphism might be independently associated with the disease susceptibility of acquired SSS

### The role of TGF-β1 gene polymorphisms in the pathogenesis of acquired SSS

T869C gene polymorphism is located in exon 1 of the TGF-β1 gene. With the T to C transition, the T869C gene polymorphism has been associated with elevated TGF-β1 serum concentrations.[17, 35] In the present study, there was no significant difference in the TGF-β1 mRNA expression levels between SSS patients and control individuals. There was also no significant difference in the TGF-β1 mRNA expression levels between CC/CT and TT genotypes of TGF-β1 gene polymorphism in the control group. The results indicated the TGF-β1 T869C gene polymorphism could not significantly affect the TGF-β1 mRNA expression. However, TGF-β1 T869C gene polymorphism has been reported to affect TGF-β1 protein trafficking or exportation efficiency of the synthesized protein to the endoplasmic reticulum. TGF-β1 T869C gene polymorphism might make serum TGF-β1 protein level increased via affecting the protein trafficking and promoting the secretion of TGF-β1 into the serum.[13, 17, 36] Accordingly, T869C gene polymorphism of TGF-β1 is suggested to modulate the serum levels for the TGF-β1 protein and subsequently leads to the dysfunction of the pacemaking activity in the fibrotic heart tissue. Thus, patients with a TGF-β1 T869C gene polymorphism might be more liable to develop acquired SSS. The present study consistently shows that the SSS group, which has a higher frequency of C at position 869, has a higher TGF-β1 serum level than the control group. This suggests a unique mechanism for the modulation of TGF-β1serum protein level, which may result in sinus node fibrosis and dysfunction.

### Study limitations

In the present study, we demonstrated that the TGF-β1 T869C gene polymorphism is associated with acquired SSS. We also provided functional results and serum TGF-β1 protein levels, to support a crucial role for TGF-β1 in the underlying pathogenesis of acquired SSS. Based on the results of PBMC mRNA level measurement and data of previous studies [13, 17, 36], they provided evidence to support that TGF-β1 T869C gene polymorphism might change the serum protein levels via affecting the protein secretion. However, we still cannot exclude the possibility of change in mRNA expression among the sinus node related cells. The potential mechanisms of the TGF-β1 that affect the susceptibility of acquired SSS via linking to the modulation of TGF-β1 protein levels in serum still need additional in vivo and in vitro studies for further clarification in the future. Another limitation of the present study is that the study population is small. These results should be further confirmed by a larger-scaled study.

## Conclusions

Patients with acquired SSS have a higher TGF-β1 T869C CC/CT genotype distribution and a higher C allele frequency, suggesting a role for the TGF-β1 T869C gene polymorphism in determining the risk of acquired SSS. The serum protein level obtained with the ELISA suggested that nucleotide substitution in the TGF-β1 polymorphic site +869 would affect the TGF-β1 serum protein levels. Taken together, the data reported in the present study provide insight for the potential mechanisms for acquired SSS. These results also suggested that the TGF-β1 T869C polymorphism is associated with high serum TGF-β1 levels and increased susceptibility to acquired SSS.

## Supporting Information

S1 Table Genotypes. (XLS)
